# Global Wave Velocity Change Measurement of Rock Material by Full-Waveform Correlation

**DOI:** 10.3390/s21227429

**Published:** 2021-11-09

**Authors:** Jing Zhou, Zilong Zhou, Yuan Zhao, Xin Cai

**Affiliations:** School of Resources and Safety Engineering, Central South University, Changsha 410083, China; zhoujing205@csu.edu.cn (J.Z.); xincai@csu.edu.cn (X.C.)

**Keywords:** structural health monitoring, coda wave interference, ultrasonic nondestructive testing, wave velocity, full-waveform

## Abstract

Measuring accurate wave velocity change is a crucial step in damage assessment of building materials such as rock and concrete. The anisotropy caused by the generation of cracks in the damage process and the uncertainty of the damage level of these building materials make it difficult to obtain accurate wave velocity change. We propose a new method to measure the wave velocity change of anisotropic media at any damage level by full-waveform correlation. In this method, the anisotropy caused by the generation of cracks in the damage process is considered. The accuracy of the improved method is verified by numerical simulation and compared with the existing methods. Finally, the proposed method is applied to measure the wave velocity change in the damage process of rock under uniaxial compression. We monitor the failure process of rock by acoustic emission (AE) monitoring system. Compared with the AE ringing count, the result of damage evaluation obtained by the proposed method is more accurate than the other two methods in the stage of increasing rock heterogeneity. These results show that the proposed method is feasible in damage assessment of building materials such as rock and concrete.

## 1. Introduction

Rock is widely used as a building material [1]. Under the action of external factors, such as load [2], temperature [3], and water [4], it will gradually be damaged and even become unstable. Monitoring and evaluating the damage of rock is an important research field. At present, nondestructive testing (NDT) methods are commonly used for structural health monitoring (SHM) and damage assessment [5]. Wave velocity reflects the internal characteristics of a medium and can be quickly and conveniently obtained by NDT with ultrasound [6] or other methods [7,8]. For these reasons, it, as an important index, is intensively applied in damage assessment.

In the field of civil engineering, ultrasonic pulse velocity is widely used in SHM and the study of the characteristics of common building materials such as concrete and rock. In the study of SHM, Mutlib et al. introduced the basic principle of the application of ultrasonic pulse velocity [9]. Ramesh et al. compared the application of different NDT technologies in SHM and pointed out that ultrasonic pulse velocity is the most versatile and a less costly method [10]. Ebrahimian et al. proposed an algorithm based on monitoring the velocity change of vertically propagating waves for structural health monitoring through the least square (LSQ) fitting identification of a beam model [11]. In the study of concrete properties, Mahdi Nematzadeh et al. characterized the mechanical properties of steel fiber-reinforced concrete containing recycled nylon granule and natural zeolite by ultrasonic pulse velocity [12]. Zhenbo Wang et al. monitored and modeled the damage evolution in engineered cementitious composite subjected to sulfate attack through the P-wave velocity, S-wave velocity, and a characteristic voltage energy parameter obtained by continuous ultrasonic measurements [13]. In the study of rock properties, Eslami et al. used continuous-wave velocity measurements during uniaxial creep tests to estimate the damage of a porous limestone [14]. Guokai Zhang et al. used P-wave velocity to study the micro- and macrocracking behaviors of flawed rocks [15]. Jingyuan Zhang et al. used the P-wave velocity to study the damage of rock caused by high temperature [16]. Jianbo Zhu et al. proposed the wave slowness ratio to describe the decay of wave velocity and linked the rock damage with the wave slowness ratio [17]. Nourani et al. used the P-wave velocity to classify and assess the rock mass parameters in Choghart iron [18]. Junwei Ma et al. used ultrasonic wave velocity tests to detect the internal microstructure of rock [19].

The wave velocity used in the above applications is calculated by the ratio of the sample length to the propagation time of the first arrival wave. This is the most common method used at present. However, building materials often do not exist alone in the field application. The cementation between concrete and rock [20], the presence of reinforcement in concrete [12], and the generation of cracks and holes [21] in the damage process render their anisotropy more significant. Guokai Zhang et al. confirmed that in the evaluation of rock damage, the assessment results obtained by the P-wave velocity measured with the first arrival wave is local evaluation rather than overall evaluation [15]. According to Fermat’s principle, the wave propagates along the shortest time. Therefore, when the wave propagates through a crack or hole in rock or concrete, it will bypass the crack or hole rather than pass through it [22]. In anisotropic media, the wave velocity measured by the first arrival wave is only the wave velocity of the single path, not the overall wave velocity of the medium. Therefore, it is unreasonable to evaluate the overall damage of the anisotropic medium.

Therefore, for anisotropic media, it is reasonable to evaluate the media damage by calculating the average wave velocity through the multipath of the wave. The coda wave interference (CWI) method proposed in recent years uses multipath to calculate the wave velocity change in media [23,24]. This method is more sensitive to the detection of weak changes in a medium because the coda wave can repeatedly sample and magnify the small changes of media [25,26]. In this method, the wave velocity change of a medium before and after the change is calculated by comparing the time-shift of the coda wave [27]. Attempts with this method have been made in the damage assessment of materials [28] such as concrete [29,30], rock [31], and steel bars [32]. Although multipath of the wave is used in CWI method to calculate the wave velocity, the theory of the CWI method assumes that the time-shift is much smaller than the dominant period [24]. This means that the CWI method cannot be used to assess the damage of a medium when the time-shift is large. Niederleithinger et al. indicated that the wave velocity change obtained by the CWI method is not meaningful anymore when the time-shift of the waveform is more than half a wavelength or the damage of the medium is large [33]. James et al. also pointed out the CWI method cannot obtain accurate assessment results when the velocity change is large [34]. In SHM or damage assessment of concrete and rock, the uncertainty of damage level and anisotropy will occur. Therefore, it is of urgency to find a method with high accuracy to obtain wave velocity change to assess different change levels of anisotropic media.

To obtain a more accurate measurement method of wave velocity change in SHM and rock or concrete damage assessment, we improve the existing methods from two aspects: time-shift solution and the relationship between time-shift and wave velocity changes. The numerical model with the different numbers of cracks is used to verify the improved method and compare the results with the onset time difference method of the first arrival wave and CWI method. A uniaxial compression experiment of red sandstone is used to verify the feasibility of the proposed method in practical application. The proposed method is used to calculate the wave velocity changes during the compression damage of red sandstone. At the same time, the AE monitoring equipment is used to collect the wave signals of crack generation during the experiment. The damage assessment results obtained by AE ringing count, the existing two methods, and the proposed method are compared.

## 2. Theory and Methodology

**Figure 1:**
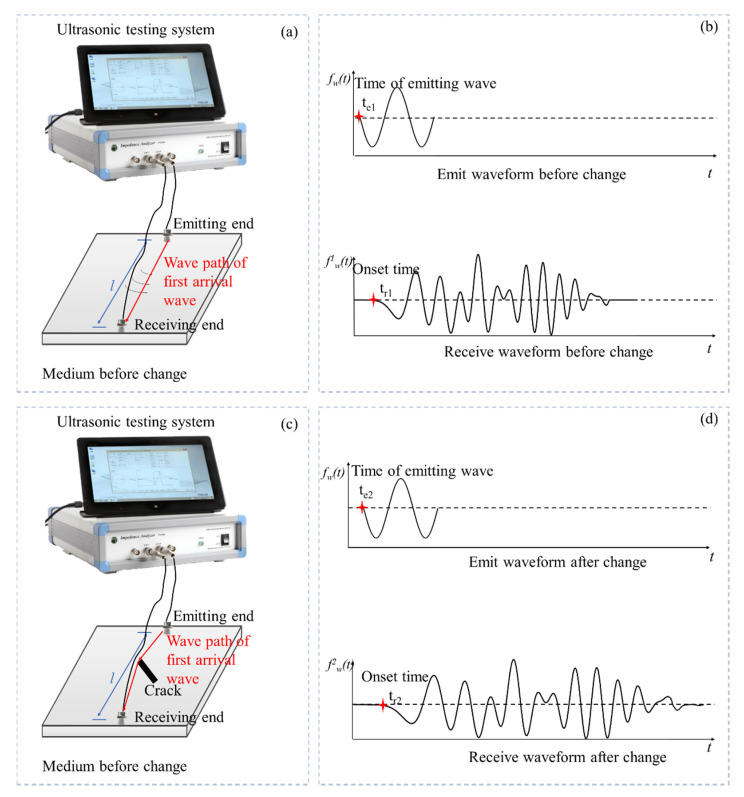
(**a**) The ultrasonic testing system and medium before the change; (**b**) the emitted and received waveform before the change; (**c**) the ultrasonic testing system and medium after the change; (**d**) the emitted and received waveform after the change.

Measuring the wave velocity change of medium by the ultrasonic testing system is shown in Figure 1a. The ultrasonic wave is emitted at one end of the detected area, and the wave propagating through the medium is received at the other end. When the medium changes, the emitting waveform remains unchanged, and the receiving waveform will change due to the change of medium, as shown in Figure 1b. Therefore, the change of medium can be evaluated by the change of the received waveform. The method proposed in our work is based on the reliability of data [35,36]. Because the reliability analysis of signal data is another research field, we do not describe it too much here. Next, we introduce how to use the correlation of full-waveform to calculate the global wave velocity change of heterogeneous media from two aspects.

### 2.1. Time-Shift Calculation

As shown in Figure 1b, the time of emitted wave and onset time of first arrival wave before the change are *t*_*e*1_ and *t*_*r*1_. These parameters after the change are *t*_*e*2_ and *t*_*r*2_. If the medium is homogeneous, the first arrival wave is the wave propagating with a straight line. We can obtain the onset time difference of the first arrival (*t_s_*) wave before and after the change as follows:

(1) $$t_{s}=t_{d2}-t_{d1}$$

where *t_d_*_2_ (*t_d_*_2_ = *t*_*r*2_ − *t*_*e*2_) is the time propagating through the medium after change, and *t_d_*_1_ (*t_d_*_1_ = *t*_*r*1_ − *t*_*e*1_) is the time propagating through the medium before change.

**Figure 2:**
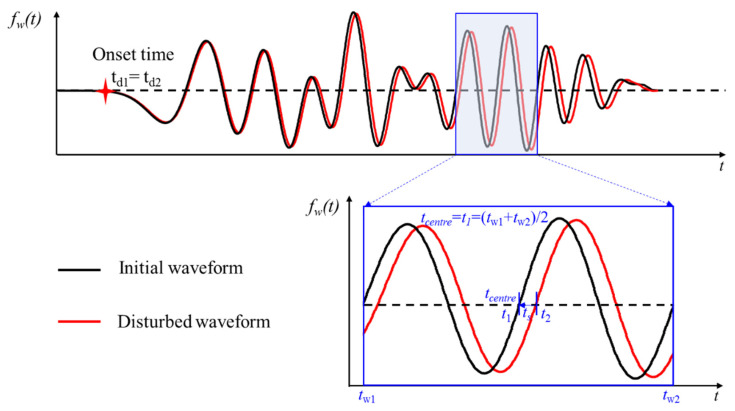
The received waveform before and after change when the medium changes slightly.

When the position of a crack is generated in the wave path of the first arrival wave, time-shift can be accurately obtained by the onset time of the first arrival wave. When the medium changes slightly, studies show that the first arrival waves will coincide, as shown in Figure 2. In this situation, it is difficult to distinguish the onset time of the first arrival waves. Figure 2 shows that the coda wave is more sensitive to the change because it can repeatedly sample in the heterogeneous medium. The repeated sampling enlarges the small change, so the coda wave can show time-shift obviously [26,37]. 

The window correlation method, trace stretching method, and dynamic time warping are three typical methods to obtain the time-shift [37,38]. These methods assume that the medium changes slightly, the first arrival wave coincides, and the coda wave is used to calculate the time-shift in this situation.

**Figure 3:**
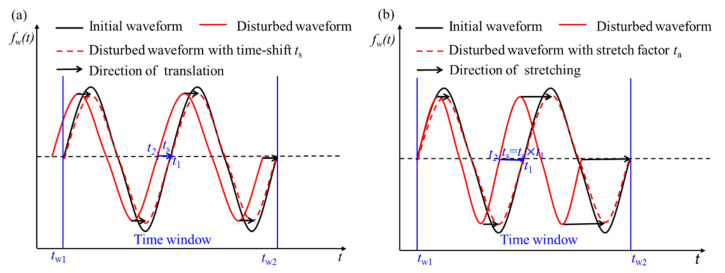
The method to obtain time-shift with translating (**a**) or stretching (**b**) the disturbed waveform on the time axis.

Figure 3 shows a time window of the initial waveform received before the change and the disturbed waveform received after the change. The method shown in Figure 3a assumes that the disturbed waveform can be translated to the initial waveform along the time axis, where *t*_*w*1_ and *t*_*w*2_ are the start and end moments of the time window. *t*_1_ and *t*_2_ (*t*_2_ = *t*_1_ + *t*_s_) are any two times with intervals of time-shift (*t*_s_) in the time window. The disturbed waveform is shifted on the time axis to make it most similar to the initial waveform; then, the time-shift is obtained.

The other method shown in Figure 3b assumes that the disturbed waveform is the stretching of the initial waveform on the time axis. From *t*_*w*1_ to *t*_*w*2_ is the range of a time window. *t*_1_ is the time of the initial waveform at the center of time window. *t*_2_ is the time of disturbed waveform at the same point with the initial waveform. Translating the disturbed waveform on the time axis cannot obtain an accurate time-shift. The time-shift is obtained when the correlation coefficient of waveform is the largest by stretching the waveform after change with stretching factor *t_a_*. Therefore, the time-shift can be expressed as *t_s_* = *t_a_* × *t*_1_.

In conclusion, the translation assumption of Figure 3a is suitable for small changes in the medium, while the stretching assumption of Figure 3b is suitable for large changes in the medium. In practice, the level of change is unknown before measurement. It is impossible to predict where the time-shift satisfies the translation hypothesis or the stretching hypothesis.

To solve this problem, the key point is to calculate the time-shift of the whole waveform. The first step is to translate and align the onset time of the first arrival wave. Then, the value and direction of the time-shift of the first arrival wave and the main period are recorded. Time-shift of the whole waveform is obtained by dividing the received waveform into several time windows with the length of twice the main period. When the change is large in a heterogeneous medium, the wave velocity and the natural vibration period of the medium will change according to the elastic wave theory [6,39]. Both translating and stretching need to be used to obtain time-shift.

**Figure 4:**
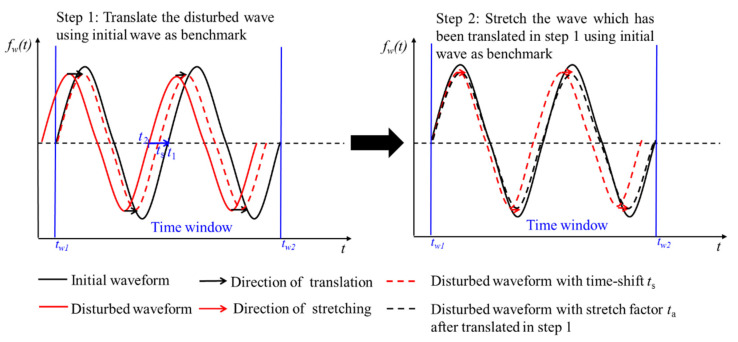
Translating and stretching are both used to calculate time-shift.

As shown in Figure 4, when time-shift is calculated in a time window, the first step is to shift the disturbed waveform to make it coincide with the initial waveform at the beginning in a time window. The second step is to stretch it to make the correlation coefficient maximum. 

The time-shift is calculated with the theory of wave field diffusion. The wave field in a medium can be regarded as the summation of all possible propagation paths [24]. The wave field of the initial medium *U_ini_* can be expressed as follows:

(2) $$U_{ini}(t)=\sum_{p}A_{p}S(t_{p})$$

where $A_{p}$ is the amplitude of the wave propagating along with the path *p*; *S*(*t_p_*) is the receive wavelet.

When the medium changes, the wave field of disturbed medium *U_dis_* can be expressed as follows:

(3) $$U_{dis}(t)=\sum_{p}A_{p}S(t_{p}+\tau_{p})$$

where $\tau_{p}$ is the travel time change of the wave propagating along with the path *p*.

In the process of calculation, the relationship *t_s_ = t_a_* × *t_*1*_ + t_b_* is used to adjust the values of translation and stretching to maximize the correlation coefficient. The correlation between the initial waveform and disturbed waveform in a time window can be expressed by correlation coefficient *R*.

(4) $$R^{\left(t_{w1},t_{w2}\right)}(t_{s})=\frac{\int_{t_{w1}}^{t_{w2}}U_{ini}(t)U_{dis}\left(t+t_{s}\right)dt}{\sqrt{\int_{t_{w1}}^{t_{w2}}U_{ini}(t)dt}\sqrt{\int_{t_{w1}}^{t_{w2}}U_{dis}(t+t_{s})dt}}$$

where *t_w_*_1_ and *t_w_*_2_ are the starting and ending moments of time window, *t_s_* is the time-shift.

The time-shift calculation of the first time window takes the value and direction of the onset time difference of the first arrival wave as the initial value. Then, the calculation of each time window takes the result of the above time window as the initial value. This ensures that accurate results of time-shift can be obtained for any change level and avoids the influence of cycle skipping.

### 2.2. Improved Relationship between Wave Velocity Change and Time-Shift

To improve calculation accuracy, the time-shift solution method proposed in Section 2.1 is necessary to establish the relationship between the wave velocity change and time-shift which is applicable for the medium changes at any level.

**Figure 5:**
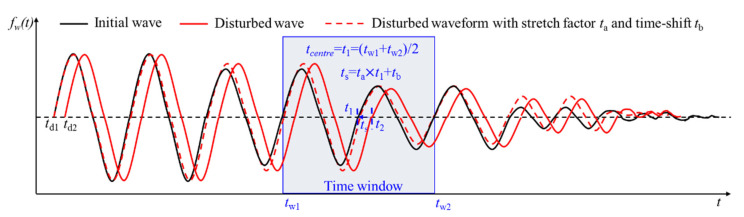
The waveforms before and after the medium change and the waveforms after stretching and translating in a time window.

Figure 5 is the initial waveforms, disturbed waveform, and the disturbed waveforms with stretching and translating i. The onset time of the first arrival wave is *t*_*d*1_ and *t*_*d*2_, respectively. *t_w_*_1_-*t_w_*_2_ is a time window and *t_centre_* is the central time of this time window. *t*_1_ is the time of initial waveform at the center time of this time window. The total time-shift in this time window is obtained by stretching *t_a_* and translating *t_b_* with the disturbed waveform. *t*_s_ ($t_{s}=t_{a}\times t_{1}+t_{b}$) is the total time-shift that makes the correlation coefficient maximum. *t*_2_ ($t_{2}=t_{1}+t_{s}$) is the time of the disturbed waveform in the time window which is determined when the correlation coefficient is maximum.

Assuming that the global wave velocity before and after the medium change is *v*_1_ and *v*_2_, respectively, and the distance between the location of the transmitting wave and the location of receiving wave is *l*, then the sum of all wave paths arriving in a time window is *L* when the correlation coefficient of the waveform is the largest. Then, we can obtain the equation $v_{1}=L/t_{1}$ and $v_{2}=L/t_{2}$.

(5) $$\frac{\Delta v}{v}=\frac{L/t_{1}-L/t_{2}}{L/t_{1}}=\frac{t_{2}-t_{1}}{t_{2}}$$

where Δv is the change of wave velocity.

The relationship between the average value of global wave velocity change and time-shift in a time window is expressed as follows.

(6) $$\frac{\Delta v}{v}=\frac{t_{s}}{t+t_{s}}$$

If only the onset time of the first arrival wave is used to calculate the wave velocity change, it can be obtained as follows:

(7) $$\Delta v=\frac{l}{t_{d1}}-\frac{l}{t_{d2}}=\frac{l(t_{d2}-t_{d1})}{t_{d1}t_{d2}}$$

(8) $$\frac{\Delta v}{v}=\frac{t_{d2}-t_{d1}}{t_{d2}}$$

If the CWI method is used to calculate the wave velocity change, the relationship between the time-shift and wave velocity change is expressed as follows [32,40]:

(9) $$\frac{\Delta v}{v}=\frac{t_{s}}{t}$$

where Δv is the wave velocity change of medium, *v* is the velocity of a medium before the change, and *t* is the moment corresponding to the center of the time window.

Comparing Equations (6), (8) and (9), the improved relationship between time-shift and wave velocity change is essentially an extension of the first arrival wave to full-waveform. Therefore, the global wave velocity change obtained with this improved relationship between time-shift and wave velocity change is more accurate in anisotropic medium.

## 3. Calculation Process

**Figure 6:**
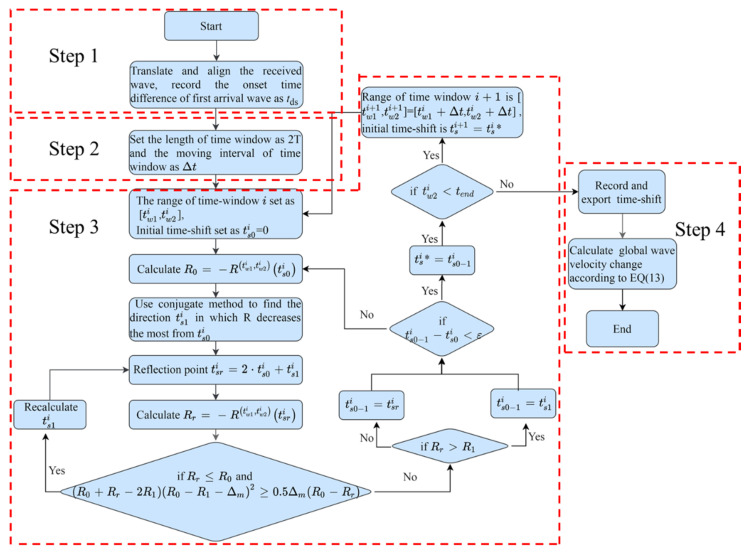
Flowchart of the improved method to calculate global wave velocity change.

The time-shift solution method and the relationship between wave velocity change and time-shift were improved in Section 2. The specific solution process is implemented by Python and expounded in Figure 6.

Step 1: Read data of the received waveforms before and after the medium change on the same time axis. Translate and align the first arrival waves. The moving time is recorded as *t_ds_*, and the onset time of the waveform is set to 0.

Step 2: Divide the initial waveform into several time windows with overlapping segments. The length of the time window is *t_l_* with a fixed value set as 2*T*. *T* is the dominant period of the disturbed waveform.

Step 3: Calculate the time-shift of each time window. The time-shift $t_{s}^{n*}$ is the value that makes the correlation coefficient R maximum. R is calculated according to Formula (4). The initial waveform in the time window is *f_w_*_1_(*t*_1_) $,\;t_{1}\in \left[\left(t_{w}^{n}-\frac{t_{l}}{2}\right),\left(t_{w}^{n}+\frac{t_{l}}{2}\right)\right]$. The waveform after the medium change is *f_w_*_2_(*t*_2_). According to the stretching and translation method proposed in Section 2, the relationship between *t*_1_ and *t*_2_ is as follows:

(10) $$t_{2}=t_{1}+\frac{t_{1}}{t_{w}^{n}}t_{s}^{n}$$

Search $t_{smax}^{n}$ with the optimization algorithm. The search range is $\left(t_{s0}^{n}-\frac{T}{2},t_{sup}^{n}+\frac{T}{2}\right)$. $t_{s0}^{n}$ is the initial value. The calculation equation is as follows:

(11) $$t_{s0}^{1}=\frac{t_{w}^{1}}{T}t_{ds}$$

(12) $$t_{s0}^{n}=\max(t_{s}^{n-1})$$

The objective function of the optimization algorithm is max [$R^{\left(t_{w}^{n}-\frac{t_{l}}{2},t_{w}^{n}+\frac{t_{l}}{2}\right)}(t_{s})$].

(13) $$R^{\left(t_{w}^{n}-\frac{t_{l}}{2},t_{w}^{n}+\frac{t_{l}}{2}\right)}(t_{s})=\frac{\int_{t_{w}^{n}-\frac{t_{l}}{2}}^{t_{w}^{n}+\frac{t_{l}}{2}}f_{w1}(t)f_{w2}(t+\frac{t}{t_{w}^{n}}t_{s}^{n})dt}{\sqrt{\int_{t_{w}^{n}-{\frac{t_{l}}{2}}_{1}}^{t_{w}^{n}+\frac{t_{l}}{2}}f_{w1}(t)dt}\sqrt{\int_{t_{w}^{n}-\frac{t_{l}}{2}}^{t_{w}^{n}+\frac{t_{l}}{2}}f_{w2}(t+\frac{t}{t_{w}^{n}}t_{s}^{n})dt}}$$

The Powell optimization algorithm [41] in SciPy toolkit is used in the optimization algorithm. This method does not need the derivation of the objective function and can also be applied when the derivative of the objective function is discontinuous.

Step 4: After searching out the $t_{s}^{n*}$ of the nth time window, record the corresponding $R_{max}^{n}$ and $t_{w}^{n}$. Then, calculate the wave velocity change rate (*k^n^*).

(14) $$k^{n}=\frac{\Delta v}{v}=\frac{t_{s}^{n*}}{t_{w}^{n}+t_{s}^{n*}}$$

Finally, the average value of wave velocity change rate of all time windows is calculated.

## 4. Verification with Numerical Simulations

### 4.1. Numerical Simulation Model

**Figure 7:**
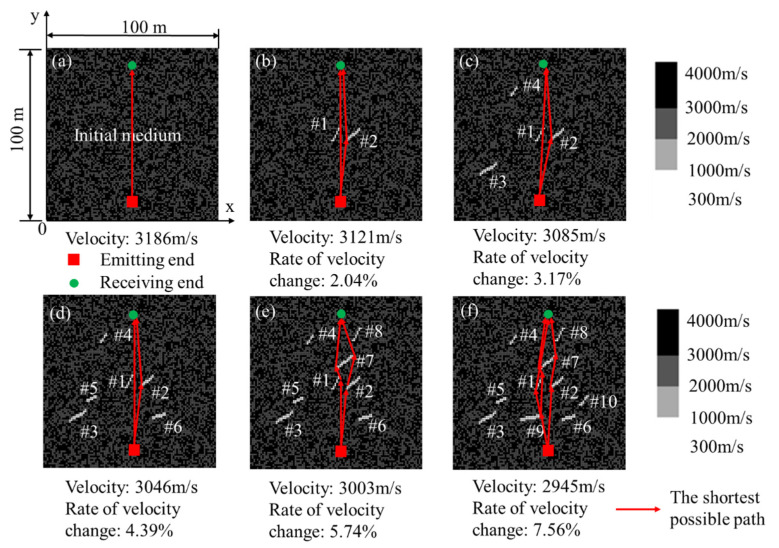
(**a**) The initial medium with no crack; (**b**–**f**) The medium with 2, 4, 6, 8, and 10 cracks.

In this section, we simulate six numerical models with the different numbers of cracks and then use the proposed method to calculate the wave velocity change of these six models. As shown in Figure 7a, the initial medium generated by the finite element method is a multiscatter medium with the size of 100 m × 100 m × 1 m. The medium is divided into 10,000 grids on average, and each grid in the model is set with different wave velocities. 

Models shown in Figure 7b–f were obtained by adding the different numbers of cracks on the initial medium. The global wave velocity and rates of wave velocity change are shown at the bottom of each figure.

**Figure 8:**
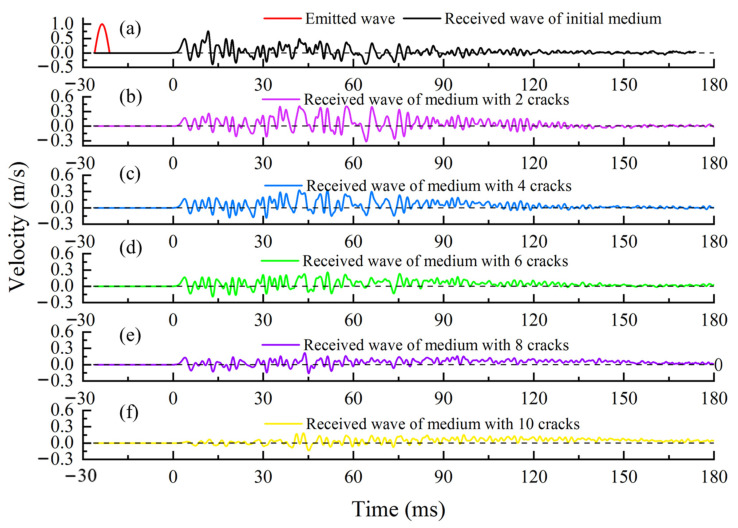
The emitted wave and the received waves of the medium with the different numbers of cracks.

To simulate the process of ultrasonic wave velocity measurement, a sine wave with a frequency of 150 kHz is shown as the red line in Figure 8. This wave is emitted at the red square areas in Figure 7. The black line and the other colored lines in Figure 8 are the received waves of medium with the different numbers of cracks. According to the theory of nondestructive testing, we can assess the global wave velocity change of the medium by analyzing the received waveform in different media.

### 4.2. Results

**Figure 9:**
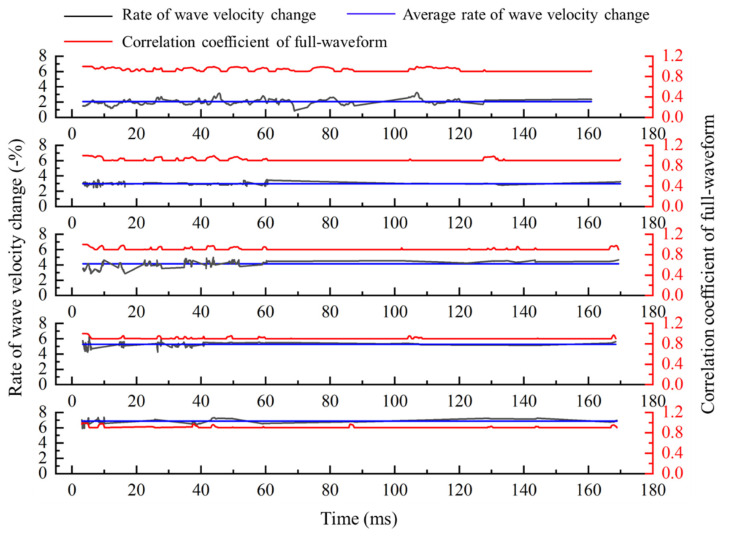
Rate of wave velocity change calculated by the method which only improves the time-shift solution.

**Figure 10:**
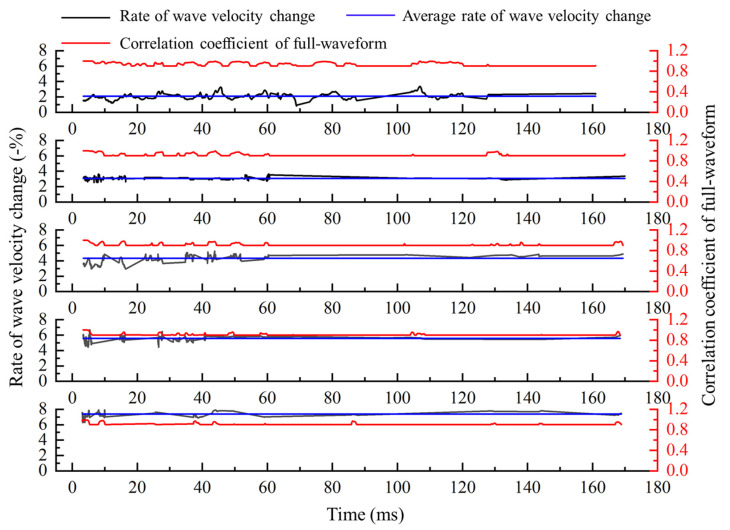
Rate of wave velocity change is calculated by the proposed method in this paper.

**Figure 11:**
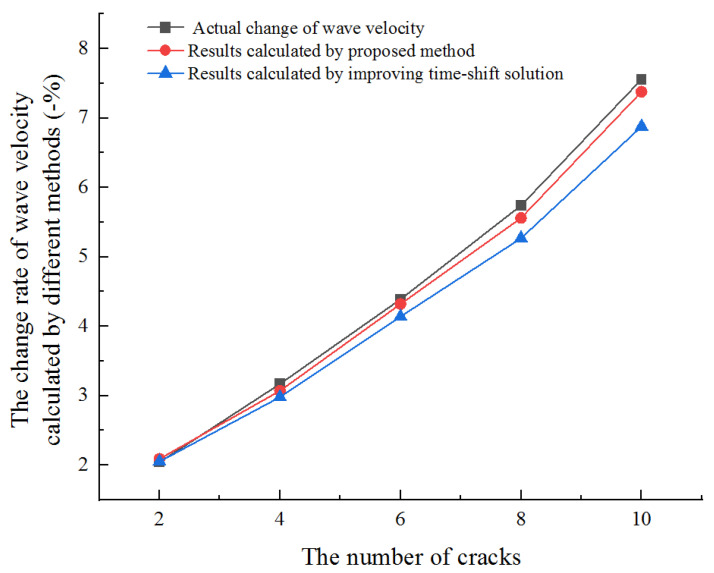
Results calculated by the proposed method in this paper and the method which only improves the time-shift solution.

We calculate the global wave velocity change according to the theory and calculation process in Section 2 and Section 3. The method with full-waveform correlation to calculate the wave velocity change is improved from two aspects. If only the solution method of time-shift is improved, the calculation results are shown in Figure 9. Both the time-shift and the relationship between time-shift and medium wave velocity change are improved; the calculation results are shown in Figure 10. From top figure to bottom figure, the results of wave velocity change calculated with only improved time-shift solution method and improved method proposed in this paper are shown in Figure 11.

As shown in Figure 11, the improved method with full-waveform correlation can obtain accurate results in the medium with the different numbers of cracks. If only the time-shift solution method is improved, the error rate increases with the increase of the crack numbers. The calculation results are accurate when the number of cracks is less than six and the error is large when the number of cracks is eight. This indicates that the two improvements proposed create a new method that can be used to measure the wave velocity change at any change level of the media.

### 4.3. Comparison with Existing Methods

In this section, we use the onset time difference method of the first arrival wave and CWI method to calculate the wave velocity change of the simulation model in Section 4.1 and compare these results with the results of the proposed method.

**Figure 12:**
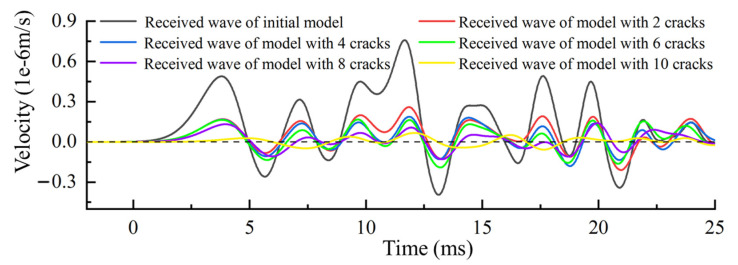
The first arrival wave before and after the change in the simulation.

Figure 12 is the first arrival wave received in the simulation. The method used to pick up the onset time of the first arrival wave is described in detail in our previous paper [42]. Then, the wave velocity change rates are calculated with the first arrival wave according to Equation (8). The results are shown in Table 1.

Results in Table 1 show that when the number of cracks in the medium are two, four, and six, the rates of wave velocity change calculated with the first arrival wave are the same. Figure 12 shows that the onset time of the first wave completely coincides with each other, and it slowly separates after half a cycle. From the relative position of the sensor emitting wave, the crack, and the sensor receiving wave, we can see that the four new added cracks, from two cracks to six cracks, in the medium are not on the linear path of ultrasonic wave emission and reception positions. According to the Fermat principle, the propagation velocity of the wave in the solid medium is much higher than that in the crack. Therefore, the four newly added cracks will not change the propagation path of the first arrival wave.

**Figure 13:**
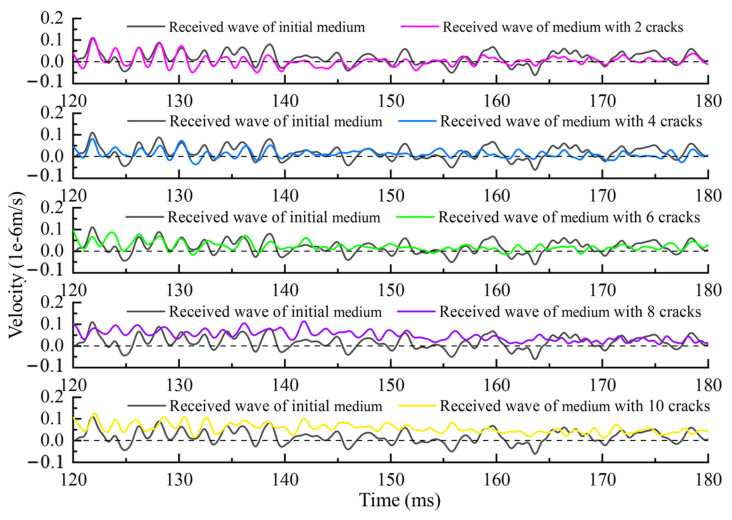
The coda wave before and after the medium change in the simulation.

The coda wave obtained in the simulation is shown in Figure 13. The black solid line is the coda wave received in the medium without cracks, and the color solid lines from top to bottom are the coda waveforms received when the number of cracks is 2, 4, 6, 8, and 10.

**Figure 14:**
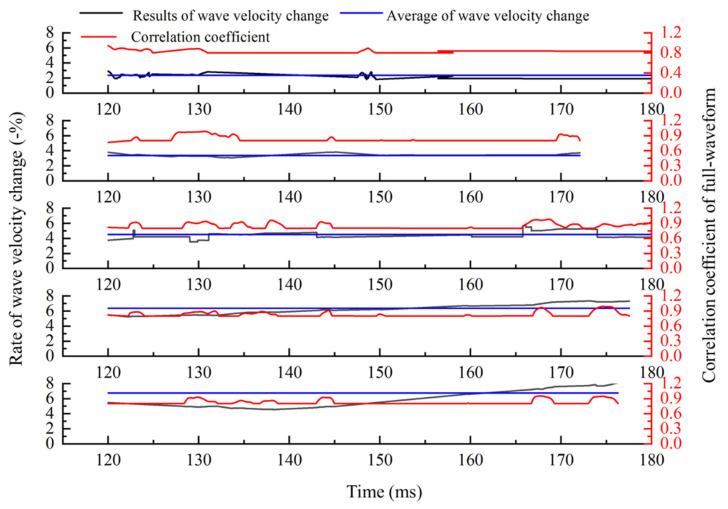
The rate of wave velocity change is calculated with the CWI method.

From top figure to bottom figure in Figure 14, the actual and calculated wave velocity change rates are shown in Table 2. Results in Table 2 show that the calculation error of CWI increases with the increase of medium change. When the medium changes greatly (larger than 5.74%), this method cannot obtain accurate results. From Figure 13, we can see that the time-shift is close to the half-period when the change rate of wave velocity is 5.74%. The time-shift will gradually increase with the increase of wave velocity change, and the CWI method is no longer applicable. This is consistent with the studies of Niederleithinger [33] and James [34].

It is difficult to directly express the accuracy of different methods to calculate the wave velocity only by the error of wave velocity change rate. Therefore, we define the error rate to compare and analyze the calculation results of different methods. The error rate of wave velocity change rate is described as follows:

(15) $$R_{error}=\frac{\left|R_{v}-R_{0}\right|}{R_{0}}$$

where *R_v_* is the calculation rate of wave velocity change with different methods, and *R*_0_ is the actual rate of wave velocity change.

**Figure 15:**
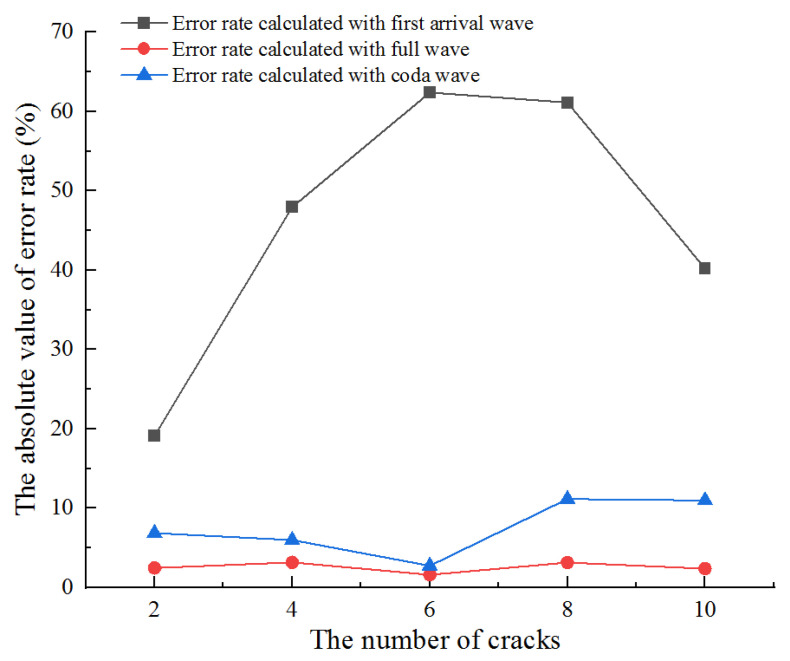
The error rate of wave velocity change rate calculated with different methods.

Results with different methods are shown in Figure 15. The results show that the error rate calculated by the onset time of the first arrival wave is the largest, especially when the number of cracks is four, six, and eight. By analyzing the location of the newly added crack, we can find that the newly added cracks are not on the linear path of ultrasonic wave emission and reception positions. Therefore, the propagation time will not change, which leads to the error of the calculation results. The cracks in the whole medium will be on the propagation path of the coda wave because it passes through multiple refraction and reflection. Therefore, the result calculated by the CWI method is relatively accurate compared with the first arrival wave. However, due to the assumption of small changes in the calculation theory of CWI, the calculation results are no longer accurate when many cracks are generated in the medium. The newly proposed method uses the correlation of full-waveform to calculate the wave velocity change, and the results are more accurate than the existing two methods. It not only overcomes the error of a single path when calculated by the first arrival wave, but also overcomes the limitations of the CWI method.

## 5. Wave Velocity Change of Rock Materials under Uniaxial Compression Experiment Calculated with the Proposed Method

### 5.1. Experiment Setup

The proposed method combining the onset time of the first arrival wave and full-waveform correlation can obtain accurate wave velocity change, which has been verified with the numerical simulations in the above contents. Next, the proposed method is used to calculate the wave velocity change of rock under the uniaxial compression experiment.

**Figure 16:**
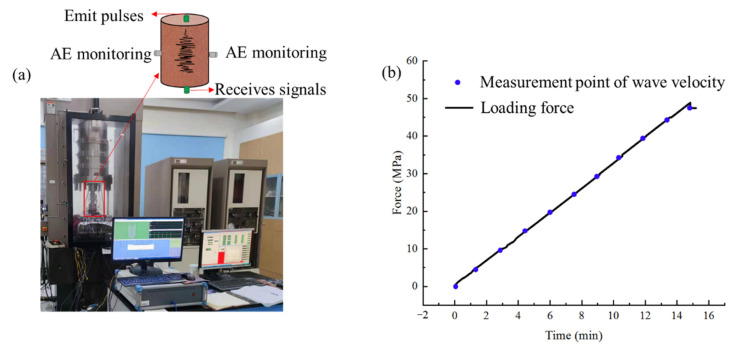
The experimental system (**a**) and loading path (**b**).

The experimental system is shown in Figure 16. A red sandstone specimen with a diameter of 50 mm and a height of 100 mm is used in this experiment. A servohydraulic rock mechanics testing system (MTS 815) is used for uniaxial compression. The loading rate of uniaxial compression is 6.4 kN/min. The AST function in the AEwinRocktest software is called to send an excitation waveform to measure the wave velocity of rock during uniaxial compression. The excitation waveform used in the test is a pulse wave with a frequency of 1 MHz. During the test, the sensor embedded in the indenter above the rock specimen emits pulses every 10 kN, and the sensor embedded in the indenter below the rock specimen receives the wave propagates along with the rock specimen. An AE acquisition system (DS5-16C) of Beijing Ruandao technology is used to monitor microfracture signals in rock uniaxial compression. The signal from the transducer is amplified by 40 dB. The sampling rate is 3 MHz.

### 5.2. Results and Discussion

**Figure 17:**
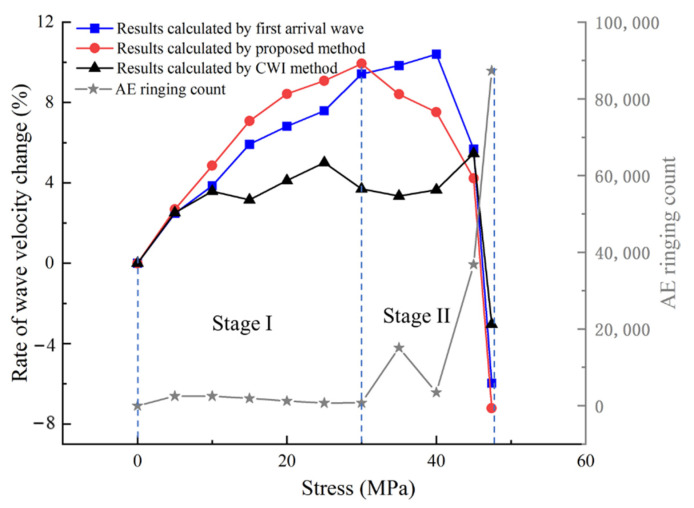
Results of wave velocity change calculated with different methods, and the AE ringing count during uniaxial compression.

The proposed method, CWI method, and first arrival wave method are used to calculate the wave velocity change during the uniaxial compression experiment. Results are shown in Figure 17. The AE ringing count is the number of new rings generated in each AST test stage.

The newly added AE ringing count in each AST test stage is generated stably when the stress is less than 30 MPa, indicating that the rock is in the compaction stage. The AE ringing count has a greater increase than the previous stage when the stress is between 30 MPa and 40 MPa, indicating that there are many microcracks generated in the rock at this stage. Then, until the rock instability stage, the AE ringing count increases sharply. It shows that many microcracks generate and extend to macrocracks, which eventually leads to rock failure.

The rate of wave velocity change calculated by the first arrival wave increases continuously with the stress. When the stress reaches 40 MPa, the rate of wave velocity change increases to the maximum value and then decreases continuously. When the rock is failed, the wave velocity is lower than the initial wave velocity of the rock specimen. The rate of wave velocity change calculated by the CWI method reaches the maximum when the stress is 45 MPa, and then decreases. The rate of wave velocity change calculated by the proposed method in this paper increases continuously with the stress. When the stress is 30 MPa, the rate of wave velocity change increases to the maximum and then decreases continuously. When the rock is destroyed, the wave velocity is lower than the initial wave velocity of the rock specimen.

Comparing the wave velocity measurement results with the AE monitoring results, the proposed method is consistent with the AE monitoring method in characterizing the damage change of rock during uniaxial compression. The change of wave velocity measured with the first arrival wave cannot represent the global damage of rock because it is the wave velocity on a single path. This phenomenon has also been pointed out in the study on the damage evolution in engineered cementitious composites subjected to sulfate attack through continuous ultrasonic measurements by Zhenbo Wang et al. [13]. The rate of wave velocity change measured by the CWI method is generally small when many AE events are generated. The results of existing research also point out that the CWI method is no longer applicable when the wave velocity of a medium changes greatly [34]. Therefore, the global change of wave velocity calculated with the proposed method is more accurate than the other two methods when the medium is anisotropic.

## 6. Conclusions

Global wave velocity change measured with NDT is conveniently and widely used in SHM and medium damage assessment. The existing onset time difference method of the first arrival wave and CWI method have some limitations because they are only applicable at some specific levels of medium change. 

To solve these problems, we improved the solution method of the time-shift by setting the translation and stretching factors and establishing an accurate relationship between the change of wave velocity and time-shift by using the wave propagation theory. Then, an improved method using full-waveform correlation to accurately measure the change of wave velocity in anisotropic media was proposed. The number of cracks in the medium was used to simulate the different levels of damage in the medium. The rates of wave velocity change of different media were calculated by the method using full-waveform, first arrival wave, and coda wave. The error rate of wave velocity change obtained by the proposed method was less than 0.83%. The results show the effectiveness of the improved method. Finally, the uniaxial compression experiment of rock specimen was carried out. The method proposed in this paper and the two existing methods were used to calculate the wave velocity change of rock specimen in the process of a uniaxial compression experiment. Then, we compared the calculation results of wave velocity change with the results of AE monitoring. The results show that the calculation results of global wave velocity change by the proposed method are more applicable in the global damage assessment of the anisotropic medium.

Overall, our study provides an accurate calculation method of global wave velocity change for SHM and media damage assessment. In future work, we will focus on the local wave velocity change calculation in anisotropic media with any change level based on this study.

## Figures and Tables

**Table 1:** The actual and calculated wave velocity change rate calculated with the first arrival wave.

	Medium with 2 Cracks	Medium with 4 Cracks	Medium with 6 Cracks	Medium with 8 Cracks	Medium with 10 Cracks
Actual value (−%)	2.04	3.17	4.39	5.74	7.56
Calculate results (−%)	1.65	1.65	1.65	2.23	4.52
Error (%)	0.39	1.52	2.74	3.51	3.04

**Table 2:** The actual and calculated wave velocity change rate calculated with the CWI method.

	Medium with 2 Cracks	Medium with 4 Cracks	Medium with 6 Cracks	Medium with 8 Cracks	Medium with 10 Cracks
Actual value (−%)	2.04	3.17	4.39	5.74	7.56
Calculate results (−%)	2.18	3.36	4.51	6.38	6.73
Error (%)	−0.14	−0.19	−0.12	−0.64	0.83

## Data Availability

The data presented in this study is available on request from the corresponding author.
